# Long-term outcome of percutaneous transluminal renal angioplasty (PTRA) versus PTRA with stenting (PTRAS) in transplant renal artery stenosis

**DOI:** 10.1186/s12872-021-02015-4

**Published:** 2021-04-26

**Authors:** Nattawut Wongpraparut, Thunyarat Chaipruckmalakarn, Thongtum Tongdee, Archan Jaspttananon, Attapong Vongwiwatana, Nalinee Premasathian, Kawin Anusonadisai, Rungtiwa Pongakasira

**Affiliations:** 1 Division of Cardiology, Department of Medicine, Faculty of Medicine Siriraj Hospital, Mahidol University, 2 Wanglang Road, Bangkoknoi, Bangkok, 10700 Thailand; 2 Interventional Radiology Unit, Department of Radiology, Faculty of Medicine Siriraj Hospital, Mahidol University, Bangkok, Thailand; 3 Division of Nephrology, Department of Medicine, Faculty of Medicine Siriraj Hospital, Mahidol University, Bangkok, Thailand; 4 Her Majesty’s Cardiac Center, Faculty of Medicine Siriraj Hospital, Mahidol University, Bangkok, Thailand

**Keywords:** Percutaneous transluminal renal angioplasty (PTRA), Percutaneous transluminal renal angioplasty with stenting (PTRAS), Transplant renal artery stenosis (TRAS), Long-term outcome

## Abstract

**Background:**

Endovascular treatment is standard of care for transplant renal artery stenosis (TRAS). No study has evaluated long-term outcomes compared between percutaneous transluminal renal angioplasty (PTRA) and PTRA with stenting (PTRAS). Accordingly, this study aimed to investigate the 1-year clinical success, and short- and long-term event-free survival between PTRA and PTRAS in patients diagnosed with TRAS at Thailand’s largest national tertiary referral center.

**Methods:**

This single-center retrospective study included kidney transplant patients treated for TRAS during January 2001 to June 2019. Clinical success was defined as (1) increase in estimated glomerular filtration rate (eGFR) > 15%, or (2) reduction in mean arterial pressure (MAP) > 15% with no decrease in antihypertensive medication, or no reduction in MAP or reduction in MAP < 15% with decrease in antihypertensive medication. Incidence of kidney transplant graft failure and transplant renal artery stenosis were also collected.

**Results:**

Sixty-five cases of TRAS were identified from 1072 patients who underwent kidney transplantation. The majority (98.5%) had end-to-side anastomosis technique. Thirty-four patients had PTRA, while 31 patients had PTRAS. One-year clinical success according to renal outcome and BP reduction was 78.5% and 49.2%, respectively. Both renal outcome (79.4% vs*.* 77.4%, *p* = 0.845) and BP reduction (40.6% vs. 58.1%, *p* = 0.166) at 1 year were similar between the PTRA and PTRAS groups. Compared between PTRA and PTRAS, event-free survival for composite of kidney transplant graft failure or transplant renal artery restenosis was significantly higher for PTRAS at 1 year (82.4% vs. 100%, *p* = 0.025), but not significantly different at 10 years (73.5% vs*.* 71%, *p* = 0.818).

**Conclusions:**

We demonstrated the 1-year clinical success, and short- and long-term event-free survival between PTRA and PTRAS in TRAS patients. One-year clinical success was found to be similar between groups. Event-free survival for composite of kidney transplant graft failure or transplant renal artery restenosis was significantly higher in PTRAS at 1 year, but similar between groups at 10 years.

*Trial registration *Thai Clinical Trials Registry, TCTR20200626002. Registered 26 June 2020—Retrospectively registered, http://www.clinicaltrials.in.th/index.php?tp=regtrials&menu=trial search&smenu = fulltext&task = search&task2 = view1&id = 6441

## Background

Organ transplantation is an essential medical advancement that can help patients return to living a normal life. The number of patients being treated by kidney transplantation (KT) in Thailand is increasing [1]. More than one thousand kidney transplants have been performed at Siriraj Hospital (Bangkok, Thailand) since the first kidney transplant at our center in 1973 [2]. Since a nationwide campaign was launched to educate the general public about the importance of organ donation, the volume of donors and recipients has increased significantly. Kidney transplantation, however, is not without potential associated complications. One such complication after kidney transplantation is transplant renal artery stenosis (TRAS), which has a reported incidence that ranges from 6 to 23% depending on the diagnostic definition [3, 4]. This complication can lead to graft loss and post-transplant hypertension. The following 3 treatment options are available for patients diagnosed with TRAS: medical therapy, percutaneous transluminal renal angioplasty (PTRA)/PTRA with stenting (PTRAS), or surgical revascularization. PTRA resulted in significantly decreased blood pressure and preserved renal function in patients with severe atherosclerotic renal artery stenosis [5]. PTRA/PTRAS is currently the standard treatment for TRAS if the lesion can be accessed using this treatment option.

Even though PTRA/PTRAS in TRAS has been studied in some retrospective reviews and a meta-analysis [6], the outcomes reported were mostly short- and mid-term outcomes 1 months to 3 years. From the systematic review performed by Ngo et al. of studies that reported outcome following PTRA and PTRAS, the majority of interventions performed were angioplasty alone in 50% of cases, with stent deployed in 37% of patients—either in combination with angioplasty or alone. Due to the small sample size that would often result from separating the two techniques, outcome of PTRA and PTRAS has commonly been reported with the two interventions combined. Too few datasets allow for formal pooled analysis to determine the efficacy of angioplasty compared to stenting [6]. The use of PTRA/PTRAS to treat TRAS was implemented at our center in 2001. The aim of this study was to investigate the 1-year clinical success, and short- and long-term event-free survival between PTRA and PTRAS in patients diagnosed with TRAS at Thailand’s largest national tertiary referral center.

## Methods

This single-center retrospective cohort study enrolled TRAS patients who underwent PTRA or PTRAS treatment at the Faculty of Medicine Siriraj Hospital, Mahidol University, Bangkok, Thailand during the January 2001 to June 2019 study period. The protocol for this study was approved by the Siriraj Institutional Review Board (SIRB) (COA no. 289/2017). This study complied with all of the principles set forth in the Declaration of Helsinki (1964) and all of its later amendments. We sought out all patients aged > 15 years who had either or both of the following International Classification of Diseases (ICD-10) codes: (1) kidney transplant status (ICD-10 Diagnosis Code Z94.0) and/or (2) renovascular hypertension (ICD-10 Diagnosis Code I15.0) with ICD 9 CM code 3950 Angioplasty of other non-coronary vessel or ICD 9 CM 3990 Insertion of non-drug-eluting peripheral vessel stent. Medical records were reviewed. Patients with TRAS who underwent PTRA or PTRAS were included. Patients with primary allograft disease, urologic complication, or infection were excluded.

We collected and recorded the patient clinical presentation that led to a diagnosis of TRAS. Non-invasive test (doppler ultrasonography, computed tomography angiography (CTA), or magnetic resonance angiography (MRA) was used for the initial investigation. The Doppler diagnostic criteria for TRAS was peak systolic velocity (PSV) 200 cm/s or increase in PSV by 50% within stenotic segment or jet aliasing [6–11]. The MRA and CTA TRAS diagnostic criteria was ≥ 50% luminal narrowing [12]. If a diagnosis of TRAS was still suspected following noninvasive diagnostic imaging, the patient was referred by his/her nephrologist for a consult with either an interventional cardiologist or an interventional radiologist, both of whom perform invasive endovascular procedures at our center. The decision regarding which type of specialist to refer the patient to was made at the discretion of the nephrologist.

All invasive endovascular procedures were performed in an inpatient setting. Risk and benefit were discussed with the patient according to standard preoperative protocol. Ipsilateral common femoral artery puncture followed by selective renal angiogram was performed in the transplanted kidney to confirm a diagnosis of TRAS if the stenosis was greater than 50%. Digital subtraction angiography (DSA) criteria was defined ≥ 50% luminal narrowing [13–16]. The treatment decision between PTRA and PTRAS was based on the severity of the lesion and the clinical judgment of the attending interventionist. In routine practice, our interventional radiologist preferred PTRA with a bailout stenting strategy, whereas our interventional cardiologist preferred routine PTRAS.

The primary endpoint was one-year clinical success after PTRA/PTRAS, which was defined, as follows: (1) Increased estimated glomerular filtration rate (eGFR) > 15% within 1 year or (2) Reduced mean arterial pressure (MAP) > 15% with no decrease in antihypertensive medication, or no reduction in MAP or reduction in MAP < 15% with decrease in antihypertensive medication within 1 year. This primary endpoint was chosen according to prior publication [6, 13, 14].

Kidney transplant graft failure was defined as failure of graft function for any reason that ultimately required renal replacement therapy and/or retransplantation [17] Transplant renal artery restenosis was suggested by rising creatinine and blood pressure, and confirmed by DSA ≥ 50% luminal narrowing [14].

### Statistical analysis

Categorical data were presented as frequency and percentage, and continuous variables were reported as mean ± standard deviation for normally distributed data or as median and interquartile range (25%, 75%) for non-normally distributed data. Categorical data were compared using chi-square test or Fisher’s exact test, and continuous data were compared using Student’s *t*-test (normality) or Mann–Whitney U test (non-normality). Event-free survival was determined using Kaplan–Meier survival analysis. A *p*-value of less than 0.05 was considered statistically significant. All statistical analyses were performed using SPSS Statistics v.18.0 (SPSS, Inc., Chicago, IL, USA).

## Results

**Fig. 1:**
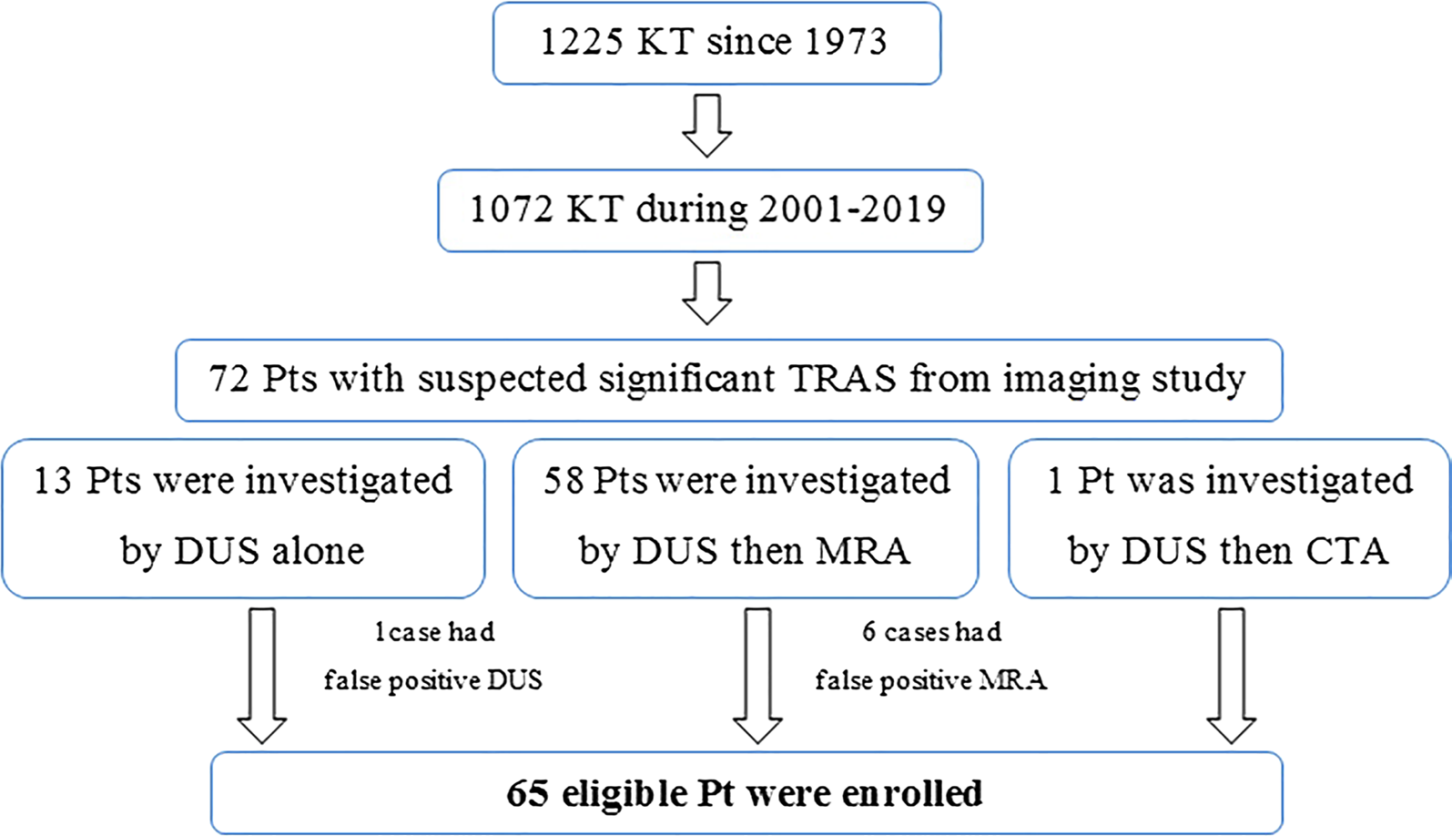
Flow diagram of the study protocol. KT, kidney transplantation; TRAS, transplant renal artery stenosis; DUS, doppler ultrasound; MRA, magnetic resonance angiography, CTA, computed tomography angiography

Our hospital’s database revealed that 1,072 kidney transplants were performed at our hospital during January 2001 to June 2019. Among those cases, 67 TRAS patients were identified (Fig. 1). This represented a 6.25% incidence rate of TRAS in this study. Two of those patients were children, so the remaining 65 adult patients were enrolled (Fig. 1).

### Baseline clinical and angiographic characteristics

Baseline clinical and angiographic characteristics are summarized in Tables 1 and 2. The mean age was 42.5 ± 11.9 years, and 61.5% of patients were men. Hypertension was the most frequent comorbidity, followed by dyslipidemia and diabetes mellitus. Regarding graft origin, 64.6% of patients received cadaveric grafts, and the remaining patients received donor kidneys from family members. Median time from transplantation to TRAS diagnosis was 3.5 months. Almost all patients in this study received end-to-side anastomosis. Anastomotic stricture (58.4%) was more common than non-anastomotic stricture (41.5%). Four of 65 patients had 2 target lesions, three of whom had anastomosis site stenosis and iliac artery stenosis, and one had anastomosis site stenosis and post-anastomosis stenosis.

**Table 1:** Patient baseline characteristics and clinical presentations compared between groups

Baseline characteristics and clinical presentations	Overall (n = 65)	PTRA (n = 34)	PTRAS (n = 31)	*p* value
Age, years	42.5 ± 11.9	42.8 ± 12.4	42.1 ± 11.5	0.817
Male gender	40 (61.5%)	21 (61.8%)	19 (61.3%)	0.969
Right ABI	1.16 (1.02, 1.28)	1.17 (1.17, 1.17)	1.15 (1.02, 1.28)	0.789
Left ABI	1.12 (0.93, 1.33)	1.17 (1.17, 1.17)	1.12 (0.93, 1.33)	0.687
ESRD etiology				
DM	7 (10.8%)	5 (14.7%)	2 (6.5%)	0.566
HT	3 (4.6%)	1 (2.9%)	2 (6.5%)	
PKD	2 (3.1%)	2 (5.9%)	0 (0.0%)	
GN	29 (44.6%)	14 (41.2%)	15 (48.4%)	
Other	24 (36.9%)	12 (35.3%)	12 (38.7%)	
Donor type				
Living	23 (35.4%)	11 (32.4%)	12 (38.7%)	0.592
Deceased	42 (64.6%)	23 (67.6%)	19 (61.3%) 19 (61.3%)	
TRAS symptomatology				
Uncontrolled HT	11 (16.9%)	6 (17.6%)	5 (16.1%)	0.987
Worsening renal function	38 (58.5%)	20 (58.8%)	18 (58.1%)	
Uncontrolled HT and worsening renal	11 (16.9%)	5 (14.7%)	6 (19.4%)	
Worsening and Pulmonary edema	1 (1.5%)	1 (2.9%)	0 (0.0%)	
Other	4 (6.2%)	2 (5.9%) 2 (5.9%)	2 (6.5%)	

Data presented as number and percentage, median (minimum,maximum), or mean ± standard deviation

PTRA, percutaneous transluminal renal angioplasty; PTRAS, percutaneous transluminal renal angioplasty with stenting; ABI, ankle brachial index; ESRD, end-stage renal disease; DM, diabetes mellitus; HT, hypertension; PKD, polycystic kidney disease; GN, glomerulonephritis

A *p*-value < 0.05 indicates statistical significance

**Table 2:** Angiographic characteristics compared between the PTRA and PTRAS groups

Angiographic characteristics	Overall (n = 65)	PTRA (n = 34)	PTRAS (n = 31)	*p* value
Time from transplant to TRAS, months	3.5 (2.1–6.0)	3.1 (2.1–6.2)	4.0 (2.2–6.0)	0.723
Time from transplant to PTRA/PTRAS, months	4.0 (3.0–7.0)	4.4 (3.0–8.0)	5.0 (3.0–7.0)	0.974
Vessel diameter (mm)	5.7 ± 3.3	5.9 ± 4.5	5.6 ± 0.9	0.672
Stenosis severity (%)	69.4 ± 17.9	67.4 ± 17.8	71.6 ± 18.1	0.343
Type of anastomosis				
End-to-side	64 (98.5%)	33 (97.1%)	31 (100.0%)	1.000
End-to-end	1 (1.5%)	1 (2.9%)	0 (0.0%)	
Location of stenosis				
Anastomosis	34 (52.3%)	17 (50.0%)	17 (54.8%)	1.000
Pre-anastomosis	1 (1.5%)	1 (2.9%)	0 (0.0%)	
Post-anastomosis	23 (35.4%)	34 (52.3%)	11 (35.5%)	
Iliac artery	3 (4.6%)	12 (35.3%)	1 (3.2%)	
Anastomosis and post-anastomosis	1 (1.5%)	2 (5.9%)	0 (0.0%)	
Anastomosis and iliac artery	3 (4.6%)	1 (2.9%)	2 (6.5%)	

Data presented as number and percentage, median (25th-75th percentiles), or mean ± standard deviation

PTRA, percutaneous transluminal renal angioplasty; PTRAS, percutaneous transluminal renal angioplasty with stenting; TRAS, transplant renal artery stenosis

A *p*-value < 0.05 indicates statistical significance

PSV and velocity gradient were both significantly reduced after intervention. Baseline and post-intervention PSV and velocity gradient were not significantly different between PTRA and PTRAS. The results of Doppler ultrasound are summarized in Table 3.

**Table 3:** Results of Doppler ultrasound compared between the PTRA and PTRAS groups

Doppler ultrasound	Overall (n = 65)	PTRA (n = 34)	PTRAS (n = 31)	*p* value
PSV				
Pre	380.6 ± 116.9	374.9 ± 115.2	386.2 ± 120.2	0.714^a^
Post	229.3 ± 78.9	225.4 ± 52.4	233.7 ± 102.2	0.789^c^
Velocity gradient				
Pre	2.86 ± 1.50	2.83 ± 1.76	2.89 ± 1.23	0.867^a^
Post	1.53 ± 0.57	1.53 ± 0.57	1.53 ± 0.57	0.618^d^
Resistive Index				
Pre	17/60 (28.3%)	10/30 (33.3%)	7/30 (23.3%)	0.352^b^
Post	12/49 (24.5%)	8/26 (30.8%)	4/23 (17.4%)	0.542^b^

Data presented as number and percentage or mean ± standard deviation

PTRA, percutaneous transluminal renal angioplasty; PTRAS, percutaneous transluminal renal angioplasty with stenting; PSV, peak systolic velocity

A *p*-value < 0.05 indicates statistical significance

^a^Independent-Sample t-test

^b^Chi-Square test

^c^Analysis of covariance (ANCOVA) was performed to assess independent differences in the level of post-PSV between group, after adjustment for pre-PSV and restenosis progression after PTRA/PTRAS

^d^Analysis of covariance (ANCOVA) was performed to assess independent differences in the level of post-Velocity gradient between group, after adjustment for pre-Velocity gradient and restenosis progression after PTRA / PTRAS

### PTRA versus PTRAS

PTRA and PTRAS were performed at a 10:9 ratio. Six patients (19%) in the PTRA group received bailout stenting. Patient data up to the last follow-up recorded in the medical record were included in our analysis. The longest follow-up was 146 months, with a median follow-up period of 54 months (interquartile range [IQR]: 25, 83 months). All patients were treated by ad hoc procedure after angiogram confirmation of TRAS diagnosis, and the procedural success rate was 100%. Minor complications occurred in 2 patients, including non-flow limited dissection in 1 patient, and hematoma at the puncture site in the other patient.

Clinical outcomes are summarized in Table 4. The 1-year clinical success rate relative to renal outcome was 78.5%. eGFR level started increasing a few days after the procedure and continued to gradually decrease over time. eGFR was not significantly different between the PTRA and PTRAS groups during the 120-month follow-up period. One-year clinical success rate relative to BP reduction was 49.2%. The PTRAS group had a similar 1-year clinical success rate relative to BP reduction when compared to the PTRA group (58.1% vs. 40.6%, *p* = 0.166). The mean systolic BP and MAP decreased immediately after successful PTRA/PTRAS, while DBP decreased at a slower pace. MAP had clinical reduction for up to 6 months after revascularization. Mean blood pressure was not significantly different between the PTRA and PTRAS groups during the follow-up period. PTRAS was shown to have superior event-free survival for composite outcomes of kidney transplant graft failure or transplant renal artery restenosis at 1 year compared to PTRA (100% vs. 82.4%, *p* = 0.025). However, event-free survival for composite outcomes of kidney transplant graft failure or transplant renal artery restenosis at 10 years was similar between PTRA and PTRAS.

**Table 4:** Clinical outcomes compared between the PTRA and PTRAS groups

Clinical outcomes	Overall (n = 65)	PTRA (n = 34)	PTRAS (n = 31)	*p* value
*One-year outcomes*				
One-year clinical success	78.5%	79.4%	77.4%	0.845
Renal outcome	49.2%	40.6%	58.1%	0.166
BP reduction	90.8%	82.4%	100%	***0.025***
One-year event-free survival for composite outcomes	95.4%	91.2%	100%	0.240
Kidney transplant graft failure	95.4%	91.2%	100%	0.240
Transplant renal artery restenosis	87.7%	88.2%	87.1%	1.000
Ten-year outcomes	72.3%	73.5%	71.0%	0.818
*Ten-year survival rate*				
Ten-year event-free survival for composite outcomes				
Kidney transplant graft failure	89.2%	91.2%	87.1%	0.701
Transplant renal artery restenosis	80.0%	82.4%	77.4%	0.619

PTRA, percutaneous transluminal renal angioplasty; PTRAS, percutaneous transluminal renal angioplasty with stenting; BP, blood pressure

Clinical success in renal outcome was defined as increase in estimated glomerular filtration rate (eGFR) > 15%. Clinical success in BP reduction was defined as reduction in mean arterial pressure (MAP) > 15% or decrease in antihypertensive medication. Composite outcome was defined as kidney transplant graft failure or transplant renal artery restenosis

A *p*-value < 0.05 indicates statistical significance

### Transplant renal artery restenosis, kidney transplant graft failure and mortality

There were 13 cases with restenosis at the target lesion, and all cases were detected due to worsening renal function or uncontrolled hypertension. Median time from revascularization to restenosis was 18.0 months (IQR: 12.1, 44.4). Three cases in the PTRA group had transplant renal artery restenosis at 4.6 months, 5 months, and 11 months, respectively. None of those 3 patients required renal replacement therapy within 1 year. Event-free survival for transplant renal artery restenosis at 1-year was 95.4%, which was not significantly different between the PTRA and PTRAS groups. Event-free survival for transplant renal artery restenosis at 10 years was 80.0%, which was also not significantly different between groups (Table 4).

**Fig. 2:**
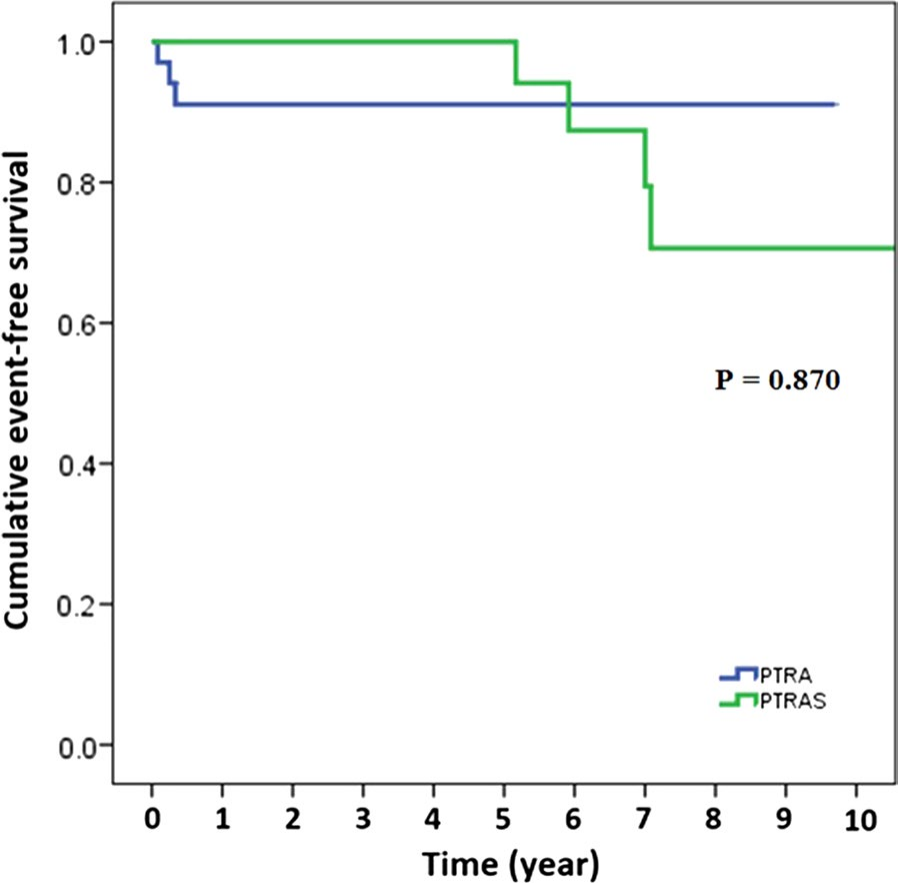
Kaplan–Meier survival estimates for kidney transplant graft failure between PTRA and PTRAS

There were 7 cases with kidney transplant graft failure. Median time from PTA to graft failure was 62 months (IQR: 3, 84). Three cases in the PTRA group had kidney transplant graft failure at 1 month, 3 months, and 4 months, respectively. Event-free survival for kidney transplant graft failure at 1-year was 95.4%, with no statistically significant difference between PTRA and PTRAS. Event-free survival for kidney transplant graft failure at 10 years was 89.2%, which was also not significantly difference between PTRA and PTRAS (Table 4). Kaplan–Meier survival curve for kidney transplant graft failure is demonstrated in Fig. 2.

**Fig. 3:**
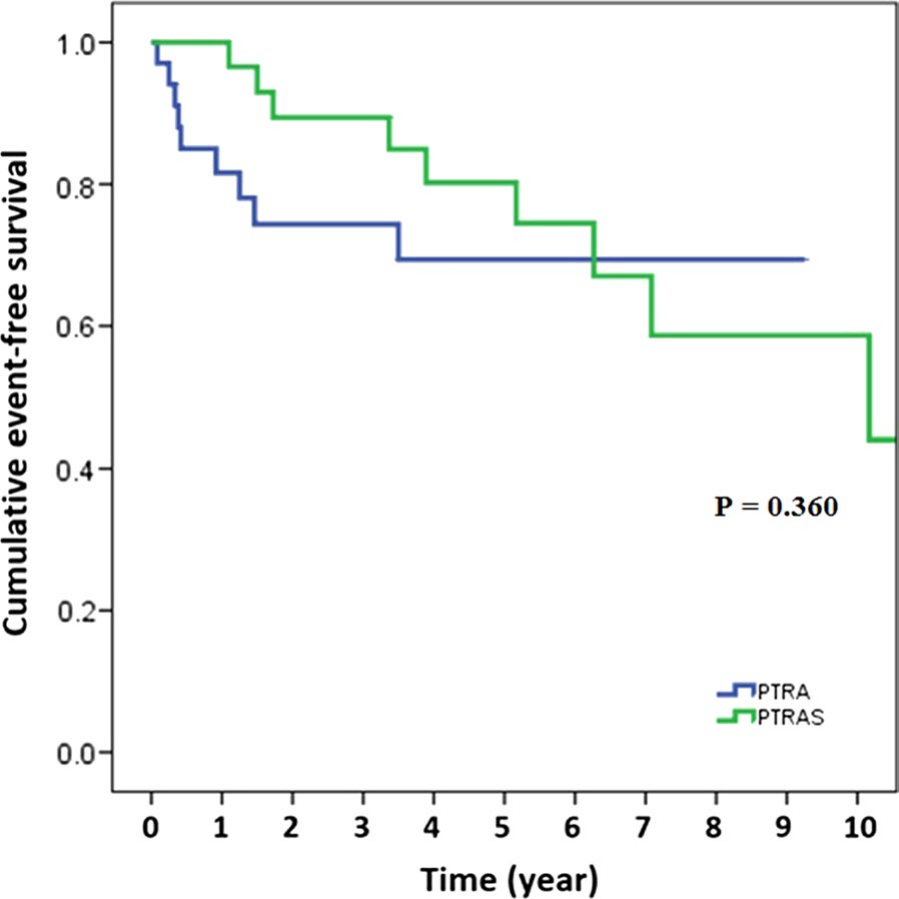
Kaplan–Meier survival estimates for composite of kidney transplant graft failure or transplant renal artery restenosis between PTRA and PTRAS

There were 6 cases in the PTRA group with kidney transplant graft failure or transplant renal artery restenosis at 1 year, while no cases of either occurred in the PTRAS group. Event-free survival for composite of kidney transplant graft failure or transplant renal artery restenosis at 1 year was 90.8%. The PTRAS group had a significantly higher event-free survival for composite of kidney transplant graft failure or transplant renal artery restenosis at 1 year than the PTRA group (100% *vs.* 82.4%, *p* = 0.025). Event-free survival for composite outcomes of kidney transplant graft failure or transplant renal artery restenosis at 10 years was 72.3%, which was not significantly different between groups (Table 4). A Kaplan–Meier survival curve for composite outcomes of kidney transplant graft failure or transplant renal artery restenosis is shown in Fig. 3.

Eight study patients died due to non-renal causes, including 5 from serious infection, 2 from malignancy, and 1 from hemorrhagic stroke. The survival rate at 10 year was 87.7%, which was not significantly different between the PTRA and PTRAS group (Table 4).

## Discussion

We found the 1-year clinical success rate to be similar between the PTRA and PTRAS group. The PTRAS group had a higher event-free survival rate for composite of kidney transplant graft failure or transplant renal artery restenosis at 1-year compared to the PTRA group. The long-term outcome, defined as kidney transplant graft failure, transplant renal artery restenosis, and all-cause mortality, was similar between the PTRA and PTRAS groups. The incidence of transplant renal artery stenosis in this study was 6.25%. Site of stenosis was most common at the anastomosis (53.8%), followed by post-anastomosis (35.4%). One-year clinical success according to renal outcome was 78.5%, and according to blood pressure reduction was 49.2%, Neither of those two outcomes were significantly different PTRA and PTRAS groups. Event-free survival for composite outcomes of kidney transplant graft failure or transplant renal artery restenosis was significantly higher in the PTRAS group at 1-year, but no significant difference between groups at 10 years.

Our study is unique in that it compared long-term 10 years outcomes between PTRA and PTRAS. Prior retrospective studies addressed the technical success, patency, and reintervention rates [6–8, 13]; however, those studies showed only mid-term outcome, with an average follow-up of around 26 months. The longest follow-up of 45 ± 35 months was reported by Voiculescu, et al. in 19 PTRA *vs.* 5 PTRAS patients. [9] Marini et al. [18] demonstrated the clinical success of PTRA with bailout stenting in 62 TRAS patients. The median follow-up was 39 months (range 1–236), and this study provided findings on allograft survival up to 10 years. Allograft survival after primary and secondary PTRA/stenting was 97% at 1-year, and 85% at 10 years. Since the strategy used was PTRA with bailout stenting (79 PTRA with 11 stents as first interventions, and 6 PTRA with 4 stents as second interventions), they did not report information comparing between PTRA and PTRAS relative to short- and long-term outcome. Due to the limited number of patients, a majority of trials pooled PTRA and PTRAS together as one intervention group. There is only limited data specific to the efficacy of angioplasty versus stenting. Our study adds information about the efficacy and long-term natural history of PTRA versus PTRAS.

Post-transplant graft loss can be caused by several reasons, including host immune response, graft rejection, primary allograft disease, infection, de novo glomerular disease, and vascular complication [19, 20]. Incidence of transplant renal artery stenosis (TRAS) was reported to range from 1 to 23% depending on the diagnostic criteria used in each study. In our study, the TRAS incidence was 6.25%, which is comparable to the 5.1% rate reported by Patil et al. [21] Allograft dysfunction was the most common presentation of TRAS in this study, which corresponds to many previous studies, including a study by Woo et al. [22] who found allograft dysfunction to be the most common indicator of TRAS. Serum creatinine level generally corresponds well with glomerular filtration rate at a certain time point, and this can be extrapolated to allograft function at that same time point. In contrast, blood pressure has many confounding factors, including activity, mood, food, and medication use. Blood pressure is, therefore, not a good diagnostic parameter for TRAS diagnosis. Timely diagnosis requires a high level of suspicion that a kidney transplant patient may have TRAS. TRAS can occur at any time after transplant. However, it usually occurs during the 3-month to 2-year post renal transplantation time period, and the most common site of stenosis is the anastomosis [23–25]. The proposed mechanism of stenosis is surgery-related fibrosis and scarring [26]. The median time from transplant to TRAS was 3.5 months in our study. Previous studies reported transplant-to-TRAS durations ranging from 1 month to 7 years. Three patients (4.6%) in this study had iliac artery stenosis. It should be noted that Doppler ultrasound if only focused at the anastomosis site cannot detect TRAS that develops in the iliac artery. Comprehensive review, including the feeding vessels, is recommended in diagnosis of TRAS.

To date, no randomized controlled studies have been conducted that establish the best context-appropriate treatments for TRAS. However, endovascular treatment has become the most frequently used method for revascularization in TRAS. PTRA/PTRAS is less invasive, has a shorter procedure duration time, allows faster recovery, and has fewer complications than surgery. PTRA was reported to be associated with a restenosis rate of 16–62%, while PTRAS was reported to have a restenosis rate of < 10%. [3, 27–29] We did not perform routine angiographic follow-up in all patients. We performed repeat renal angiogram in patients with clinically-driven suspected for transplant renal artery restenosis, such as worsening renal function or uncontrolled blood pressure. This may explain the lower restenosis rate in this study. PTRAS showed a significantly higher event-free survival rate for composite outcomes of kidney transplant graft failure or transplant renal artery restenosis at 1 year, but no significant difference was observed between PTRAS and PTRA at 10 years. Stent at the anastomosis site could cause abnormal flow and increased shear stress resulting in late intimal hyperplasia and neoatherosclerosis. In the PTRA group, if the graft survived through the first year, vessel patency remained excellent.

The majority of TRAS in our study were stenosis at the anastomosis site, which suggests pathophysiology due to surgery-related fibrosis and scarring at the anastomosis site. This explains why only PTRA had a favorable long-term outcome in our study. This study addressed the advantages and disadvantages over the long-term between PTRA and PTRAS. Since TRAS supply the only kidney, the first strategy of treatment is PTRAS to reassure the patency of the remaining vessels. The second strategy is PTRA with bailout stenting if no major dissection is required, there is only < 20% residual stenosis, and there is no acute vessel closure. Stenting could be avoided. The second strategy may increase the risk of vessel closure, future restenosis, and renal transplant graft failure. In the present study, 6 of 34 patients in the PTRA group had either kidney transplant graft failure or transplant renal artery restenosis at 1 year. PTRAS had a higher event-free survival for composite of kidney transplant graft failure or transplant renal artery restenosis at 1 year, but event-free survival for composite of kidney transplant graft failure or transplant renal artery restenosis at 10-year was similar between the PTRAS and PTRA groups.

### Study limitations

This was a retrospective study. We did not directly compare between PTRA and PTRAS using a prospective randomized trial design, but we used the procedural entity to separate the group. Bias of referral pattern to specialist and specialist procedural preferences cannot be excluded. There existed the potential for more procedural complexity, more challenging lesion, and more unsatisfactory angiographic outcome after PTRA in the PTRAS group. Even though initial degree of renal artery stenosis between PTRA and PTRAS was not significantly different, we cannot exclude selection bias between two groups. We used only bare metal stent in all cases included in this study. Few studies reported a better outcome with drug-eluting stent, especially in arteries less than 5 mm in diameter [30]. However, a majority of patients in our study has average vessel diameter > 5 mm. For long-term vessel patency, we used clinically-driven restenosis. We did not perform re-angiogram in every patient. In setting of transplant renal artery stenosis when renal function greatly depended on transplant kidney. Significant restenosis affecting renal blood flow would represent with allograft dysfunction. However, we cannot exclude mild or moderate restenosis that may have occurred between groups.

This study did not show a significant difference in long-term outcome between PTRA and PTRAS in TRAS. However, our small sample size may have limited the statistical power of our study to identify all significant differences and associations between groups. Sample size required 213 patients per group for sufficient statistical power, and we were only able to identify a total of 65 patients.

## Conclusion

Endovascular treatment is a preferred treatment for TRAS. Due to the limited number of patients undergoing this procedure, there is no long-term outcome for PTRA versus PTRAS. We demonstrated similar 1-year clinical success between PTRA and PTRAS in TRAS patients; however, PTRAS was shown to confer superior event-free survival for composite outcome of kidney transplant failure or transplant renal artery restenosis at 1-year, but the results between groups were similar at 10 years. A larger-scale prospective multicenter study is needed to more accurately determine the outcome differences between PTRAS and PTRA in TRAS.

## Data Availability

The datasets used and/or analyzed during the current study are de-identified and available from the corresponding author on reasonable request. Identifying/confidential patient data should not be shared.

## Abbreviations

PTRA: Percutaneous transluminal renal angioplasty
PTRAS: Percutaneous transluminal renal angioplasty with stenting
TRAS: Transplant renal artery stenosis
eGFR: Estimated glomerular filtration rate
MAP: Mean arterial pressure
KT: Kidney transplantation
DSA: Digital subtraction angiography
PSV: Peak systolic velocity
